# Phytoconstituents of *Artemisia annua* as potential inhibitors of SARS CoV2 main protease: an *in silico* study

**DOI:** 10.1186/s12879-024-09387-w

**Published:** 2024-05-15

**Authors:** Eraj Irfan, Erum Dilshad, Faisal Ahmad, Fahad Nasser Almajhdi, Tajamul Hussain, Gholamreza Abdi, Yasir Waheed

**Affiliations:** 1 Department of Bioinformatics and Biosciences, Faculty of Health and Life Sciences Capital, University of Science and Technology, (CUST), Islamabad, Pakistan; 2 Foundation University Medical College, Foundation University Islamabad, Islamabad, 44000 Pakistan; 3 Present Address: COVID-19 Virus Research Chair, Botany and Microbiology Department, College of Science, King Saud University, 11451 Riyadh, Saudi Arabia; 4 Center of Excellence in Biotechnology Research, King Saud University, 11451 Riyadh, Saudi Arabia; 5 Department of Biotechnology, Persian Gulf Research Institute, Persian Gulf University, Bushehr, 75169 Iran; 6 Gilbert and Rose-Marie Chagoury School of Medicine, Lebanese American University, Byblos, 1401 Lebanon; 7 MEU Research Unit, Middle East University, Amman, 11831 Jordan; 8 Present Address: Near East University, Operational Research Center in Healthcare, TRNC Mersin 10, Nicosia, 99138, Turkey

**Keywords:** SARS-CoV-2, *Artemisia annua*, CB-dock, Chrysoplenetin, Azithromycin

## Abstract

**Background:**

In November 2019, the world faced a pandemic called SARS-CoV-2, which became a major threat to humans and continues to be. To overcome this, many plants were explored to find a cure.

**Methods:**

Therefore, this research was planned to screen out the active constituents from *Artemisia annua* that can work against the viral main protease Mpro as this non-structural protein is responsible for the cleavage of replicating enzymes of the virus. Twenty-five biocompounds belonging to different classes namely alpha-pinene, beta-pinene, carvone, myrtenol, quinic acid, caffeic acid, quercetin, rutin, apigenin, chrysoplenetin, arteannunin b, artemisinin, scopoletin, scoparone, artemisinic acid, deoxyartemisnin, artemetin, casticin, sitogluside, beta-sitosterol, dihydroartemisinin, scopolin, artemether, artemotil, artesunate were selected. Virtual screening of these ligands was carried out against drug target Mpro by CB dock.

**Results:**

Quercetin, rutin, casticin, chrysoplenetin, apigenin, artemetin, artesunate, sopolin and sito-gluside were found as hit compounds. Further, ADMET screening was conducted which represented Chrysoplenetin as a lead compound. Azithromycin was used as a standard drug. The interactions were studied by PyMol and visualized in LigPlot. Furthermore, the RMSD graph shows fluctuations at various points at the start of simulation in Top1 (Azithromycin) complex system due to structural changes in the helix-coil-helix and beta-turn-beta changes at specific points resulting in increased RMSD with a time frame of 50 ns. But this change remains stable after the extension of simulation time intervals till 100 ns. On other side, the Top2 complex system remains highly stable throughout the time scale. No such structural dynamics were observed bu the ligand attached to the active site residues binds strongly.

**Conclusion:**

This study facilitates researchers to develop and discover more effective and specific therapeutic agents against SARS-CoV-2 and other viral infections. Finally, chrysoplenetin was identified as a more potent drug candidate to act against the viral main protease, which in the future can be helpful.

**Supplementary Information:**

The online version contains supplementary material available at 10.1186/s12879-024-09387-w.

## Background

SARS-CoV-2 first emerged in China and then transmitted to the rest of the world. It first emerged in bats and was later transmitted to humans with an intermediatory source that is still not known [1]. The virus belongs to the β-group and is a positive-sense RNA-enveloped virus. This zoonotic virus can easily adapt and become more virulent with time [2]. The diameter ranges from 65–125 nm and has four main structural proteins, which include spike glycoproteins, membrane glycoproteins, small envelope glycoproteins and nucleocapsid protein with several other proteins [3]. The life cycle of the virus depends upon two main polypeptides pp1a and pp1ab which further process 15 non-structural proteins with the help of papain-like protease and the main protease M^pro^ [4]. This main protease is responsible for cleavage at 11 sites in the replicase protease. For this reason, M^pro^ is selected as a potential drug target site [5]. M^pro^ a 33.8 KDa CoV enzyme plays a major role by digesting the replicase polyproteins of the virus at 11 conserved sites. This makes M^pro^ an efficient drug target site against the active constituents present in *Artemisia annua* [6]. This protease is considered a cysteine protease as it has a cysteine histidine catalytic dyad and cleaves peptide bonds at Glm-Ser/ Ala/Gly [7]. In SARS-CoV-2 14 different proteolytic sites of PLpro and 3CLpro were determined. At the N-terminal, PLpro cleaved three sites at 181–182, 818–819 and 2763–2764 and at the C-terminal it cleaves 11 different sites producing 15 non-structural proteins. These contain Nsp3 with multiple domains with a unique domain of SARS-a proteolytic enzyme and a deubiquitylation enzyme. Nsp5 is the 3CLpro, Nsp12 is RdRp (RNA dependent-RNA polymerase) and Nsp13 is a helicase [8].

The virus-infected individuals showed variations in symptoms and the rate of infection. Human coronavirus caused upper respiratory tract infections [9]. An initial study showed that almost 91% of affected individuals showed high fever, 77% had a cough, 44% individuals felt fatigued, other symptoms like salivation were shown by 28%, headache by 8%, 5% had hemoptysis and 3% faced diarrhea [10]. For the treatment of the symptoms shown, four kinds of vaccines have been developed, which include the whole virus vaccine, second one is the type of recombinant protein subunit vaccine which specifically targets the spike protein. The replication-incompetent vaccine and the nucleic (mRNA) based vaccine [11]. Many drugs were also repurposed, azithromycin has been commonly used in Pakistan and other countries. Azithromycin is used during respiratory, urinary, dermal and other bacterial infections. It is also used during chronic inflammatory disorders that include post-transplant bronchitis, diffuse pan bronchitis and rosacea [12]. Many medicinal plants such as Ar*temisia annua* belonging to the family Asteraceae were also exploited. This plant is rich in around 600 active metabolites and has shown anti-fungal, anti-asthmatic, hepatoprotective and antioxidant properties [13]. In Madagascar, a drink was prepared with the infusion of *Artemisia annua* and other plants to cure Covid-19 [14].

In addition, the development of antiviral medications that impede the activity of the SARS-CoV-2 Mpro is viable and has potential for practical use. Phytochemicals produced Ar*temisia annua* aid in combating illnesses caused by fungus, bacteria, and plant viruses [15]. The rationale for selecting this plant is based on the phytochemicals they contain generally have beneficial effects on health [16]. This include bioactive nutrients that have been shown to have positive effects on human health by reducing the risk of major chronic illnesses. Phytochemicals have been shown to have potential efficacy in treating many illnesses, as indicated by preclinical, clinical, and epidemiological studies, owing to their antioxidant and anti-inflammatory properties [17].

Molecular docking has been in use for the past three decades for drug designing through computer assistance. Docking is preferred while performing virtual screening for the analysis of the functions of the compounds. Results can easily be classified through docking and it can give a detailed analysis of how the ligand interacts with the protein, which can optimize the lead compounds for drug development [18]. Different docking programs use one or more search programs for prediction of possibilities of receptor-ligand complexes. For this purpose, molecular docking has become a key tool for drug discovery and molecular modeling applications. The result gives a score of the interaction, making it more reliable for predicting the ligand pose and, through that pose binding site of the ligand can easily be determined [19].

## Materials and methods

### Retrieving protein’s structure

For the study, the SARS CoV-23 CLpro or M^pro^ was selected as a potential drug target site. The crystal structure of the main protease was downloaded under the PDB ID 6lu7 (https://www.rcsb.org/structure/6lu7).

### Cleaning of protein’s structure

M^pro^ is a linear chain that consists of 1–306 amino acids referring to its A chain. Extra constituents were present as side chain C and the Nitrogen and water components were removed through Pymol [20].

### Determining the physicochemical properties of M^pro^

The physicochemical properties of the protein which are molecular weight, isoelectric point, amount of negative and positive residues, extinction coefficient, instability index, aliphatic index and GRAVY were determined using ProtParam, which is a tool of ExPAsy [21].

### Ligand selection and preparation

The reported antiviral ligands of *Artemisia annua* were selected as potential hits. The 3D structures for the 25 selected ligands were downloaded from the PubChem database (https://pubchem.ncbi.nlm.nih.gov/). The data was obtained in SDF format. The 3D structures were represented in Kekule’s form. The molecular mechanics 2 (MM2) energy of the ligands was minimized by the use of Chem3D ultra [22]. The reported compounds compactness was removed with addition of hydrogen atoms and making them flexible against the target protein. This was followed by drug likeness and lead likeness by applying Lipinski rule of five and other filters using SwissAdme [23, 24].

### Molecular docking

A blind docking software CB-Dock [25] was used for the docking of proteins and ligands. It finds the docking sites automatically and gives the calculation of the size, center and sites of bonding in 5 different poses of interaction. A grid box of x, y and z coordinated were adjusted i.e. x = -27.77, y = 13.56, z = 56.09 with size of x = 51.4, y = 62.45 and z = 53.84. The best pose is the one with the minimum vina score in KJ/mol.

### Visualization of docking result

For visualization of the docked results, Pymol was used which provides a plugin that can access results easily and makes their visualization clear.

### Analysis of docking result

For the 2D generation of protein–ligand complex LigPlot has been used which provides an analysis of hydrophobic and hydrogen bond interactions.

### ADME and drug likeness studies

By using the PkCSM pharmacokinetics tool ADME + T properties (Absorption, Distribution, Metabolism + Toxicity) were studied and toxicity was determined of the leading compounds (Quercetin, rutin, catechin, chrysoplenetin, apigenin, artemetin, artesunate, sopolin and sitogluside) and the comparative drug (Azithromycin) [26].

The druglikeness was studied using (SwissADME server http://www.swissadme.ch/) Lipinski rule of 5 (octanol–water partition coefficient log *P* value of drug like compound be limited to 5, molecular weight should be > 500, hydrogen bond acceptance number be 10 and hydrogen bond donor number should be 5) [24].

### Molecular dynamics simulations

Molecular Docking Studies can predict ligand binding states in static conditions. Docking provides a static depiction of how a chemical interacts with the active site of a protein [27]. To compute atom motions over time, MD simulations employ Newton's classical equation of motion. Amber16 software was applied for the complex system to check the carbon alpha atom structural deviation throughout the time scale [28]. They can predict ligand binding status in physiological environments [29, 30]. Protein Preparation was done using chimera with 750 steepest and conjugate gradients with a total of 1500 steps to perform complicated optimization and minimization on the receptor-ligand combination. Both the systems were prepared against the Top1 and Top2 complex system using the System Builder tool. TIP3P (Transferable Intermolecular Interaction Potential 3 Points) of AMBER16 tool was chosen as a solvent model with an orthorhombic box. To neutralize the models, counter ions were introduced. 0.15 M sodium chloride (NaCl) was added to mimic physiological circumstances. The NPT ensemble with a temperature of 300 K and a pressure of 1 atm was utilized throughout the simulation. Before running the simulation, the models were relaxed. The trajectories were collected every 200 ps, and the system was prepared by introducing minimization, heating, equilibrium and pressure states. Final production of the simulation has been run via Amber 16 and RMSD (root mean square deviation) of the protein and ligand over time was used to test the stability of the simulations. The total simulations time given for both the systems was applied for 100 ns. Followed by trajectories analysis with RMSD, RMSF, SASA, Hydrogen bonds analysis, PCA and Binding free energies calculations.

## Results

### Refining of protein

**Fig. 1:**
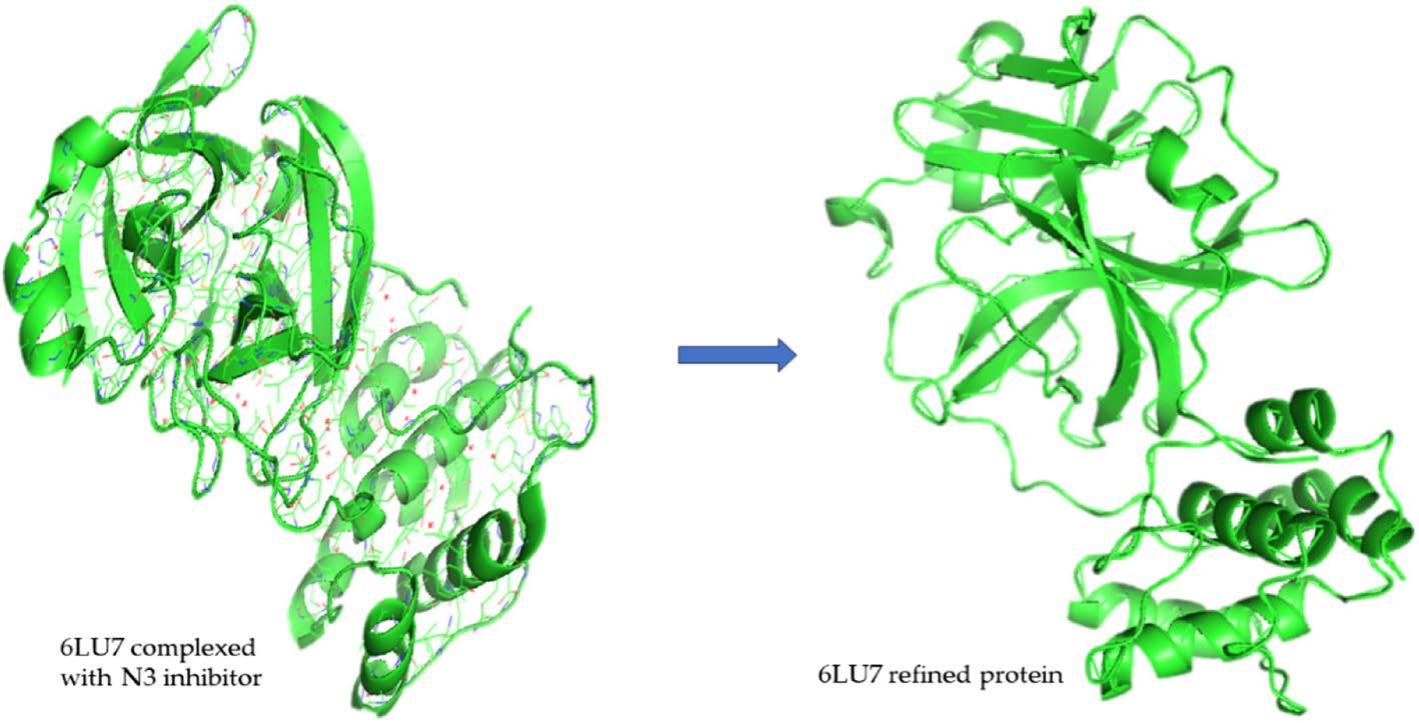
Refining of 6LU7

The 3D structure was obtained from PDB (Protein Data Bank) under the name 6LU7 with the DOI number 10.2210/pdb6LU7/pdb [31]. The structure obtained was attached with an N3 inhibitor, through PyMol the inhibitor and the extra side chain C was removed as shown in Fig. 1. Domains I and II had an anti-parallel β barrel structure, whereas Domain III has a globular structure consisting of 5 anti-parallel α helices. Domain III was connected by Domain II by a loop region consisting of 185–200 residues [6].

### Physicochemical properties of protein

By using ProtParam a tool of ExPASy the physiochemical properties were studied. Table 1 shows the collective weight of negative and positive amino acid residues as 33,796.64. The pI value indicates acidic nature of M^pro^. The low value of GRAVY indicates good interactions with water molecules and values for instability index and aliphatic indexes show the stability of the protein.

**Table 1:** Physical properties of M^pro^

MW	pI*	NR*	PR*	
33,796.64	5.95	26	22	
Ext. Co 1	Ext. Co 2	Instability Index	Aliphatic Index	GRAVY
33,640	32,890	27.65	82.12	-0.019

^*^MW Molecular Weight, ^*^pI Isoelectric Point, ^*^NR negatively charged residues, ^*^PR positively charged residues

### Identification of functional domains of protein

Functional domains are the active part of the protein that are used for interaction with other proteins and substances. The InterPro job ID for finding the functional domains of 6LU7 is https://www.ebi.ac.uk:443/interpro//result/InterProScan/iprscan5-R20210417-071019-0353-62313319-p2m/. Two promoters A and B combine to form a 306 amino acid long polypeptide consisting of three domains. Domain I is 8–101 residues, Domain II is 102–184 residues and Domain III are 201–303 residues. There is a cleft between domain I and II which acts as a binding site [6].

### Selection of ligands

Ligand selection was based on the best resolution structure, chemical class of the crystals bound to the protein and binding affinities. The most important is the conformation of the ligand. The active constituents of the selected plant were searched from the world’s largest chemical databank, i.e. PubChem [14, 24, 32–34]. From PubChem 25 active constituents of *Artemisia annua* based on resolution, chemical class, binding affinity and conformations, the 3D structures were downloaded in SDF format. The energies of the downloaded structures were minimized using MM2 force field in chem draw 3D software so that no effect on the docking score occurs. The selected ligands include Alpha-pinene, Beta-pinene, Carvone, Myrtenol, Quinic acid, Caffeic acid, Quercetin, Rutin, Apigenin, Chrysoplenetin, Arteannunin b, Artemisnin, Scopoletin, Scoparone, Artemisnic acid, Deoxyartemisnin, Artemetin, Casticin, Sitogluside, Beta-sitosterol, Dihydroartemisnin, Scopolin, Artemether, Artemotil and Artesunate (Table S2) [13, 14, 32, 33, 35].

### Drug-likeliness and toxicity prediction

Drug likeness of the compounds was done by Lipinski Rule of Five which indicates that the molecular weight of a protein should be ≤ 500, log P ≤ 5, H-bond donors ≤ 5, and H-bond acceptors ≤ 10. The rules are followed by orally administered drugs. A compound following three of the rules is considered a drug [24, 36]. Supplementary Table S1 shows that Rutin does not follow Lipinski rule whereas Sitogluside disobeys two of the rules.

#### Toxicity prediction

PkCSM [37] an online tool was used to check the toxicity of the ligands and that of the standard drug. Table 2 shows the results of toxicity prediction. The table shows that both artemisinin and dihydroartemisinin are AMES toxic which means that they can be mutagenic and can later be carcinogenic. Rutin and beta-sitosterol are hERG II inhibitors that can lead to the potassium channel inhibition leading to QT syndrome. Myrtenol and artemisinic acid are sensitive to skin. The values of *T.pyriformis* toxicity show that β-pinene, artemisinic acid and scopoletin are toxic. Sitogluside and beta-sitosterol are minimally toxic.

**Table 2:** Toxicity prediction

S.No	Ligands	AMES Toxicity	Max. tolerated dose (human)	hERG I inhibitor	hERG II inhibitor	Oral rat acute toxicity	Oral rat chronic toxicity	Hepatotoxicity	Skin sensitization	*T. pyriformis* toxicity	Minnow toxicity
1	α-pinene	No	0.48	No	No	1.77	2.262	No	No	0.45	1.159
2	β-pinene	No	0.371	No	No	1.673	2.28	No	No	0.628	1.012
3	Carvone	No	0.775	No	No	1.86	1.972	No	Yes	0.41	1.445
4	Myrtenol	No	0.439	No	No	1.746	1.8	No	Yes	0.262	1.698
5	Quinic acid	No	1.626	No	No	1.128	3.529	No	No	0.285	4.869
6	Caffeic acid	No	1.145	No	No	2.383	2.092	No	No	0.293	2.246
7	Quercetin	No	0.499	No	No	2.471	2.612	No	No	0.288	3.721
8	Rutin	No	0.452	No	Yes	2.491	3.673	No	No	0.285	7.677
9	Casticin	No	0.47	No	No	2.302	1.768	No	No	0.317	2.233
10	Chrysoplenetin	No	0.491	No	No	2.324	1.773	No	No	0.313	2.248
11	Apigenin	No	0.328	No	No	2.45	2.298	No	No	0.38	2.432
12	Arteannunin b	No	0.195	No	No	2.052	1.589	No	No	0.45	1.53
13	Artemisinic acid	No	0.403	No	No	1.747	2.251	No	Yes	0.541	0.541
14	Artemisinin	Yes	0.065	No	No	2.459	1	No	No	0.322	1.406
15	Deoxyartemisinin	No	0.174	No	No	2.161	1.506	No	No	0.363	1.538
16	Dihydroartemisinin	Yes	0.014	No	No	2.227	0.995	No	No	0.298	1.067
17	Artemetin	No	0.335	No	No	2.36	1.025	No	No	0.332	1.842
18	Artemether	No	0.074	No	No	2.429	1.043	No	No	0.304	0.587
19	Artemotil	No	0.019	No	No	2.32	0.952	No	No	0.347	1.799
20	Artesunate	No	0.256	No	No	3.112	1.549	No	No	0.285	1.499
21	Scopoletin	No	0.614	No	No	1.95	1.378	No	No	0.516	1.614
22	Scoparone	No	0.494	No	No	2.345	2.408	No	No	0.603	1.223
23	Scopolin	No	0.393	No	No	2.391	3.756	No	No	0.286	4.198
24	Sitogluside	No	-0.887	No	No	2.571	3.293	No	No	0.285	-0.811
25	Beta-sitosterol	No	-0.621	No	Yes	2.552	0.855	No	No	0.43	-1.802

### Molecular docking

Molecular docking was performed using M^pro^ as receptor protein and the ligands selected above. The protein in PDB format and the ligands in SDF format were docked. The CB dock server was applied and then check the input files by converting them into pdbqt format files using OpenBabel and MGL Tools [38, 39]. Then CB dock predicts the cavities of the receptor and calculates the centers and sizes of the top five cavities. Among the five best conformations the best one was selected based on a high-affinity score of the interaction between the protein and the ligand [40]. Ligands showing the best binding score between the selected ligands and the protein M^pro^ are shown in Table 3.

**Table 3:** Molecular docking results of the selected compounds against the target protein

S.No	Ligands	Binding Score Kc/mol	Cavity size	HBD*	HBA*	logP	Molecular Weight g/mol	Rotatable Bonds	Grid Map
1	α-pinene	-4.8	212	0	0	2.9987	136.23	0	53.705
2	β-pinene	-4.7	212	0	0	2.9987	136.23	0	53.705
3	Carvone	-5.1	212	0	1	2.4879	150.22	1	53.705
4	Myrtenol	-5.2	212	1	1	1.9711	152.23	1	53.705
5	Quinic acid	-5.4	258	5	5	-2.3214	192.17	1	71.716
6	Caffeic acid	-6	212	3	3	1.1956	180.16	2	53.705
7	Quercetin	-7.6	258	5	7	1.988	302.23	1	71.716
8	Rutin	-6.9	258	10	16	-1.6871	610.5	6	71.716
9	Casticin	-7.6	258	2	8	2.9056	374.3	5	71.716
10	**Chrysoplenetin**	**-7.7**	**258**	**2**	**8**	**2.9056**	**374.3**	**5**	**71.716**
11	Apigenin	-7.8	258	3	5	2.5768	270.24	1	71.716
12	Arteannunin b	-6.6	212	0	3	2.4518	248.32	0	53.705
13	Artemisinic acid	-7	212	1	1	3–6458	234.33	2	53.705
14	Artemisinin	-7	212	0	5	2.3949	282.33	0	53.705
15	Deoxyartemisinin	-7.2	258	0	4	2.4633	266.33	0	71.716
16	Dihydroartemisinin	-7.1	212	1	5	2.1867	284.35	0	53.705
17	Artemetin	-7.6	258	1	8	3.2086	388.4	6	71.716
18	Artemether	-7.1	212	0	5	2.8408	289.37	1	53.705
19	Artemotil	-7.1	258	0	5	3.2309	312.4	2	71.716
20	Artesunate	-7.5	258	1	7	2.6024	384.4	4	71.716
21	Scopoletin	-5.9	212/258	1	4	1.5072	192.17	1	53.705/71.716
22	Scoparone	-6	212	0	4	1.8102	206.19	2	53.705
23	Scopolin	-7.5	258	4	9	-1.0197	354.31	4	71.716
24	Sitogluside	-7.6	688/239	4	6	5.849	576.8	9	56.178/58.474
25	Beta-sitosterol	-6.9	212	1	1	8.0248	414.7	6	53.705
26	**Refrence Drug (Azithromycin)**	-6.8	156	5	14	4.76	748.98	7	53.537

^*^HBD Hydrogen bond donor, ^*^HBA Hydrogen bond acceptor

Ligands like quercetin, rutin, casticin, chrysoplenetin, apigenin, artemetin, artesunate, scopolin and sitogluside have shown good docking results. Rutin shows a binding score of -8.9 kJ/mol with a log *P* value of -1.6871. Apigenin shows a binding score of -7.8 kJ/mol with a log *P* value of 2.5768. chrysoplenetin shows a binding score of -7.7 kJ/mol and logP vale is 2.9056. Quercetin, Artemetin, casticin and sitogluside show vina score as -7.6 kJ/mol with log *P* values as 1.988, 3.2086, 2.9056 and 5.849 respectively. Scopolin and artesunate show a score of -7.5 kJ/mol with log *P* values as -1.0197 and 2.6024. Ligands like quercetin, rutin and apigenin had already been reported to be docked against M^pro^ by using Auto dock wizard as reported by Oluwaseun Taofeek in 2020. Quercetin showed a score of -7.2 which is less than the docking score by CB-dock. Rutin showed a score of -7.7 kJ/mol and apigenin gave a binding score of -6.8 kJ/mol which is less than that shown by CB-dock [41].

### Interaction of ligands and protein

**Fig. 2:**
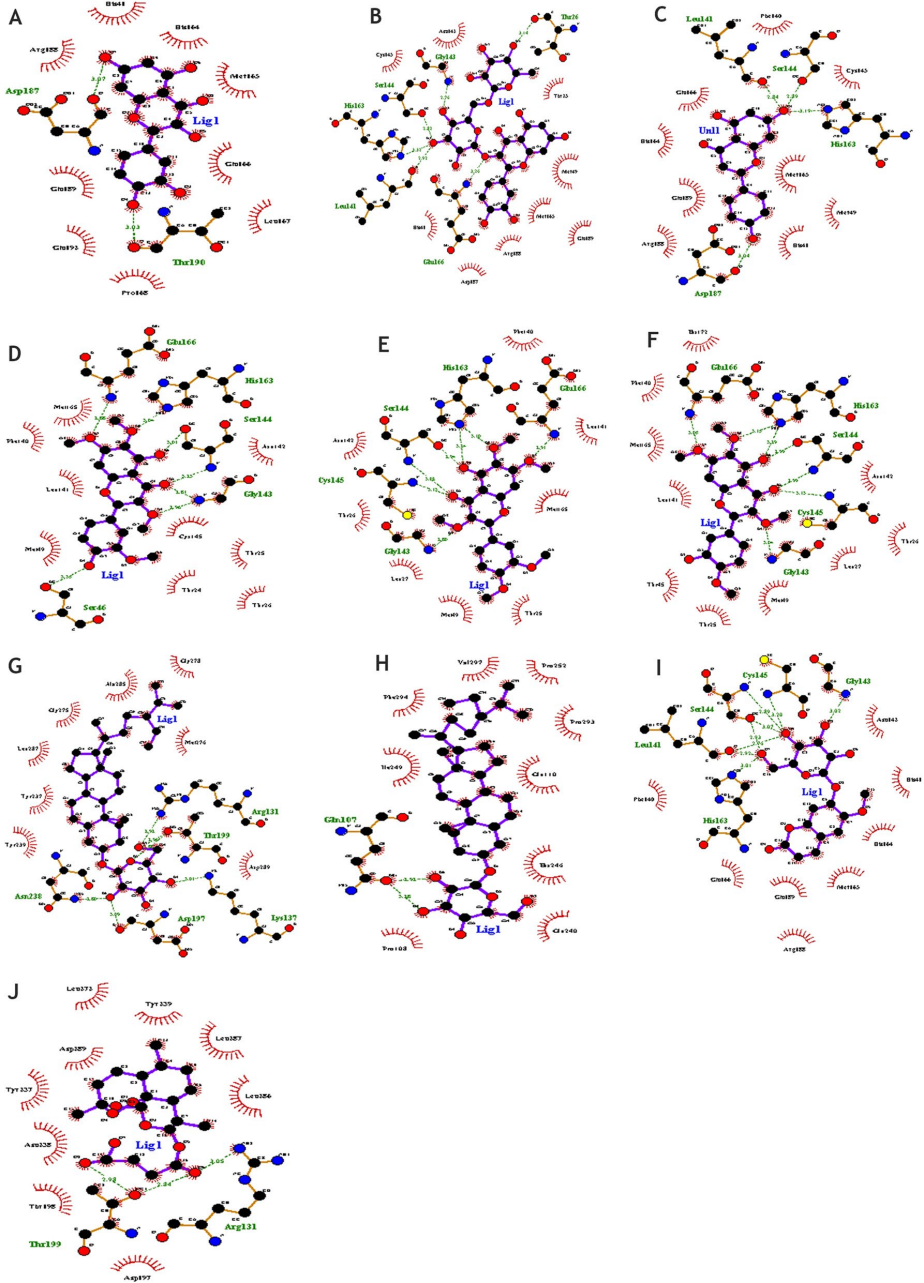
**A** Interaction of quercetin with M^pro^, **B** Interaction of rutin with M^pro^, **C** Interaction of apigenin with M^pro^, **D** Interaction of chrysoplenetin with M^pro^, **E** Interaction of artemetin with M^pro^, **F** Interaction of casticin with M^pro^, **G** Interaction of sitogluside at cavity 1 with M^pro^, **H** Interaction of sitogluside at cavity 4 with M^pro^ (**I**) Interaction of scopolin with M^pro^ and (**J**) 2 J Interaction of artesunate with M^pro^

The deducted docked results were analyzed using LigPlot and PyMol. The interaction between the ligands and the receptor protein was predicted through LigPlot + [42]. The graphical system of LigPlot automatically generates 2D pictures of interactions from its 3d coordinates. The 2D diagrams of the interaction of the ligands with the best docking score and the standard drug with the protein are shown in Fig. 2a-J. Whereas Table 4 shows the hydrogen and hydrophobic interactions.

**Table 4:** Hydrogen and hydrophobic interactions

S.No	Ligand Name	Binding Energy (Kcal/mol)	No.of Hydrogen Bonds	Hydrogen Bonding		Hydrophobic Bonding
				**Amino Acids**	**Distance**	
**1**	**Quercetin**	-7.6	2	O-Thr190-O4 O-Asp187-O7	3.03 3.07	Arg188 His41 His164 Met165 Glu166 Leu167 Pro168 Gln192 Gln189
**2**	**Rutin**	-6.9	6	N-Glu166-O2 O-Leu141-O15 NE2-His163-O15 OG-Ser144-O15 N-Gly143-O16 O-Thr26-O12	3.26 2.92 3.32 2.82 2.76 3.14	Thr25 Met49 Gln189 Met165 Arg188 Asp187 His41 Cys145 Asn142
**3**	**Apigenin**	-7.6	4	O-Leu141-O4 OG-Ser144-O4 NE2-His163-O4 O-Asp187-O5	2.84 2.89 3.19 3.04	Phe140 Cys145 Met49 Met165 His41 Arg188 Gln189 His164 Glu166
**4**	**Chrysoplenetin**	-7.7	7	OG-Ser46-O4 N-Gly143-O5 N-Gly143-O2 N-Ser144-O2 OG-Ser144-O6 NE2-His163-O8 N-Glu166-O7	3.35 2.96 2.81 3.23 3.01 3.04 2.88	Asn142 Cys145 Thr25 Thr24 Thr26 Met49 Leu141 Phe140 Met165
**5**	**Artemetin**	-7.6	7	N-Glu166-O7 N-Gly143-O5 N-Cys145-O2 N-Ser144-O2 OG-Ser144-O6 NE2-His163-O6 NE2-His163-O8	2.91 2.80 3.13 3.02 2.94 3.24 3.10	Phe140 Leu141 Met165 Thr25 Met49 Leu27 Thr26 Asn142
**6**	**Casticin**	-7.6	7	N-Gly143-O5 N-Cys145-O2 N-Ser144-O2 OG-Ser144-O6 NE2-His163-O6 NE2-His163-O8 N-Glu166-O7	3.04 3.13 2.99 2.99 3.29 3.15 3.05	Asn142 Thr26 Leu27 Met49 Thr25 Thr45 Leu141 Met165 Phe140 His172
**7**	**Sitogluside**	-7.6–1	5	NH2-Arg131-O3 OG1-Thr199-O3 NZ-Lys137-O4 O-Asp197-O6 ND2-Asn238-O6	2.92 2.96 3.01 3.09 2.80	Tyr239 Tyr237 Leu287 Gly275 Ala285 Gly278 Met276 Asp289
		-7.6–4	2	OE1-Gln107-O6 OE1-Gln107-O5	2.92 3.28	Ile249 Phe294 Val297 Pro252 Pro293 Gln110 His246 Glu240 Pro108
**8**	**Scopolin**	-7.5	8	NE2-His163-O7 O-Leu141-O7 O-Leu141-O9 OG-Ser144-O7 OG-Ser144-O9 N-Ser144-O9 N-Cys145-O9 N-Gly143-O8	3.01 2.92 2.76 2.93 3.07 2.89 3.20 3.02	Asn142 His41 His164 Met165 Gln189 Arg188 Glu166 Phe140
**9**	**Artesunate**	-7.5	3	NH2-Arg131-O6 OG1-Thr199-O6 OG1-Thr199-O8	3.05 2.84 2.98	Leu272 Tyr239 Leu287 Leu286 Asp197 Thr198 Asn238 Tyr237 Asp289

### ADME properties of ligands

pkCSM is the second tool used for the assessment of ADME properties [18].

#### Absorption

Table S3 shows the result of absorption properties of ligands. Quercetin, rutin, sitogluside, scopolin and artesunate show low CaCO_2_ solubility. Rutin shows poor intestinal absorption. All ligands act as P-glycoprotein substrates. Artemetin and sitogluside act as P-glycoprotein I inhibitors and artemetin, sitogluside, casticin and chrysoplenetin act as P-glycoprotein II inhibitors. Azithromycin shows a low CaCO_2_ solubility and water solubility. It has a lower value of skin permeability and is a P-glycoprotein substrate and P-glycoprotein I inhibitor. The Pkcsm absorption properties of artemetin, casticin, scopolin, and artesunate have already been reported by Zarina Khurshid in 202. The absorption parameters of quercetin, rutin and apigenin have been studied by Oluwaseun Taofeek in 2020 [41].

#### Distribution

Distribution properties (Table S4) show that quercetin, casticin, scopolin and rutin cannot cross the blood brain barrier. Quercetin, rutin, casticin, scopolin, artesunate and chrysoplenetin are not CNS permeable. Azithromycin shows a low VDss value which means the drug would not be properly distributed. The distribution parameters of quercetin, rutin and apigenin have already been studied by Oluwaseun Taofeek [23] and that of artemetin, scopolin, artesunate and casticin have been reported by Zarina Khurshid [40].

#### Metabolism

All the ligands were not CYP2D6 substrates, chrysoplenetin, artemetin, casticin, sitogluside and artesunate were all CYP3A4 substrates. Quercetin, apigenin, chrysoplenetin, artemetin and casticin were all CYP1A2 inhibitors. Apigenin, chrysoplenetin, artemetin and casticin were CYP2C19 inhibitors. Only artemetin was a CYP2C9 inhibitor. All the ligands were not CYP2D6 inhibitors, chrysoplenetin and casticin were CYP3A4 inhibitors (Table 5). Pkcsm metabolic properties of scopolin, artesunate, artemetin and casticin have been reported by Zarina Khurshid [40]. Some of the metabolism parameters of quercetin, rutin and apigenin have been recorded by Oluwaseun Taofeek [41].

**Table 5:** Metabolism properties of the ligands and standard drug

S.No	Ligands	CYP2D6 substrate	CYP3A4 substrate	CYP1A2 inhibitor	CYP2C19 inhibitor	CYP2C9 inhibitor	CYP2D6 inhibitor	CYP3A4 inhibitor
1	Quercetin	No	No	Yes	No	No	No	No
2	Rutin	No	No	No	No	No	No	No
3	Casticin	No	Yes	Yes	Yes	No	No	Yes
4	Chrysoplenetin	No	Yes	Yes	Yes	No	No	Yes
5	Apigenin	No	No	Yes	Yes	No	No	No
6	Artemetin	No	Yes	Yes	Yes	Yes	No	No
7	Artesunate	No	Yes	No	No	No	No	No
8	Scopolin	No	No	No	No	No	No	No
9	Sitogluside	No	Yes	No	No	No	No	No
10	Azithromycin (Standard Drug)	No	Yes	No	No	No	No	No

#### Excretion

Table 6 indicates that all the ligands and standard drugs are not renal OCT2 substrates which means that they would not be cleared out from the body.

**Table 6:** Excretion properties of the ligands and standard drug

S.No	Ligands	Total Clearance	Renal OCT2 Substrate
1	Quercetin	0.407	No
2	Rutin	-0.369	No
3	Casticin	0.628	No
4	Chrysoplenetin	0.627	No
5	Apigenin	0.566	No
6	Artemetin	0.706	No
7	Artesunate	0.969	No
8	Scopolin	0.716	No
9	Sitogluside	0.689	No
10	Azithromycin (Standard Drug)	-0.424	No

Pkcsm excretion properties of scopolin, artesunate, artemetin and casticin have been reported previously [40]. Quercetin, rutin, and apigenin have been recorded by Oluwaseun Taofeek [41].

### Lead compound identification

After the first knockout by Lipinski’s rule and the second by the study of pharmacokinetics rutin was knocked out. Other ligands based on docking score and properties were taken to the next level. After that chrysoplenetin was selected as the lead compound depending upon its pharmacokinetics and pharmacodynamics.

### Comparison of standard drug with lead compound

The lead compound chrysoplenetin was compared with the standard drug azithromycin and their physiochemical and pharmacokinetic properties were compared for the assessment of bioavailability, safety, efficiency, and drug-likeness.

#### Lipinski’s rule comparison

The comparison shows that azithromycin breaks two of Lipinski’s rules that are of molecular weight and H-bond acceptor as the molecular weight exceeds the limit of 500, and the H-bond acceptance value exceeds 10. The lead compound however follows all the given Lipinski’s rules (Table S4).

#### ADMET properties comparison

##### Toxicity comparison

The value of toxicity parameters of Azithromycin shows that this drug can be toxic to liver, but other parameters are in the range of positive values. Azithromycin cannot cause any sensitivity to the skin and is also not an inhibitor of hERG I and hERG II. The dose value of 1.927 is also tolerable. With that a no to AMES toxicity indicates that it is not carcinogenic.

The toxicity comparison is based on 9 models (Table 7). Model I of AMES toxicity shows that none of these are mutagenic, Model II shows that chrysoplenetin has a low value of dose. The third model shows that both are not hERGI and hERGII inhibitors. The fourth and fifth model of oral rat acute and chronic toxicity value give the value of a low dose that could result in an adverse effect. The hepatotoxicity model shows that azithromycin is hepatotoxic. Model seven indicates that neither of the two are skin sensitive. Toxicity model 8 and 9 indicate that azithromycin is somewhat toxic.

**Table 7:** Toxicity properties comparison

S.No	Model Name	Azithromycin	Chrysoplenetin
1	AMES Toxicity	No	No
2	Max. tolerated dose (human)	1.927	0.491
3	hERG I inhibitor	No	No
4	hERG II inhibitor	No	No
5	Oral rat acute toxicity	2.769	2.324
6	Oral rat chronic toxicity	1.991	1.773
7	Hepatoxicity	Yes	No
8	Skin sensitization	No	No
9	*T. pyriformis* toxicity	0.285	0.313
10	Minnow toxicity	7.8	2.248

##### Absorption properties comparison

The parameter of absorption is based on 6 models (Table 8). CaCO_2_ solubility suggests that chrysoplenetin is absorbed more than azithromycin. Intestinal absorption of azithromycin is also low. Chrysoplenetin is a P-glycoprotein II inhibitor whereas azithromycin is a P-glycoprotein I inhibitor.

**Table 8:** Absorption properties comparison

S. No	Reference drug	Azithromycin	Chrysoplenetin
1	**Water Solubility**	-4.133	-3.605
2	**CaCO**_**2**_** Solubility**	-0.211	1.393
3	**Intestinal Absorption (human)**	45.808	99.856
4	**Skin Permeability**	-2.742	-2.743
5	**P-glycoprotein substrate**	Yes	Yes
6	**P-glycoprotein I inhibitor**	Yes	No
7	**P-glycoprotein II inhibitor**	No	Yes

##### Metabolic properties comparison

Metabolic properties indicate that azithromycin is a CYP3A4 substrate whereas chrysoplenetin is a CYP3A4 substrate and an inhibitor to CYP1A2, CYP2C19 and CYP3A4 (Table 9).

**Table 9:** Metabolic properties comparison

S. No	Reference Drug	Azithromycin	Chrysoplenetin
1	**CYP2D6 substrate**	No	No
2	**CYP3A4 substrate**	Yes	Yes
3	**CYP1A2 inhibitor**	No	Yes
4	**CYP2C19 inhibitor**	No	Yes
5	**CYP2C9 inhibitor**	No	No
6	**CYP2D6 inhibitor**	No	No
7	**CYP3A4 inhibitor**	No	Yes

##### Distribution properties comparison and PAINS alert

Table 10 shows that azithromycin has poor distribution to the brain, and it will not be able to pass the central nervous system. Further it has been investigated for PAINS alert as well to check whether the compounds interact with human proteins inadvertently or not. Upon investigating both compounds 0 PAINS alert were inferred showing no interactions with human protein targets.

**Table 10:** Distribution properties comparison

S. No	Reference Drug	Azithromycin	Chrysoplenetin
1	**VDss (human)**	-0.214	-0.161
2	**Fraction unbound (human)**	0.512	0.103
3	**BBB Permeability**	-1.857	-1.043
4	**CNS Permeability**	-3.777	-3.226

##### Excretion properties comparison

The excretion properties of both the compounds are in the safe range. Chrysoplenetin has more total clearance than azithromycin (Table 11).

**Table 11:** Excretion properties comparison

S. No	Reference Drug	Azithromycin	Chrysoplenetin
1	**Total Clearance**	-0.424	0.627
2	**Renal OCT2 Substrate**	No	No

#### Physicochemical properties comparison

This screening tells that azithromycin has 38 carbon atoms, 72 hydrogen atoms, 2 nitrogen atoms and 12 oxygen atoms whereas chrysoplenetin has 19 carbon atoms, 18 hydrogen atoms and 8 oxygen atoms. The number of atoms indicates that chrysoplenetin is a simple bio-compound in comparison to azithromycin. Azithromycin can donate 5 hydrogen atoms whereas chrysoplenetin can donate 2 hydrogen atoms showing the oxidation state of each. Azithromycin accepts 14 hydrogen atoms which are against Lipinski’s rule. The molecular weight of azithromycin also exceeds the rule limit of 500 g/mol. Chrysoplenetin has 5 rotatable bonds while azithromycin has 7 rotatable bonds (Table 12).

**Table 12:** Physiochemical properties comparison

S. No	Drug	Molecular Formula	H-bond donor	H-bond acceptor	Log P-value	Molecular Weight g/ mol	Rotatable Bonds
1	**Azithromycin**	C_38_H_72_N_2_O_12_	5	14	1.9007	748.996	7
2	**Chrysoplenetin**	C_19_H_18_O_8_	2	8	2.9056	374.3	5

#### Docking score comparison

Both the standard drug and lead compound were docked against M^pro^. Table 13 shows the docking results which indicate that the binding score of azithromycin is -6.8 and that of chrysoplenetin is -7.7. The result indicates that chrysoplenetin can block or bind with M^pro^ more efficiently than azithromycin.

**Table 13:** Docking score comparison

S.No	Compound	Binding Score
1	Azithromycin	-6.8
2	Chrysoplenetin	-7.7

#### Docking analysis comparison

**Fig. 3:**
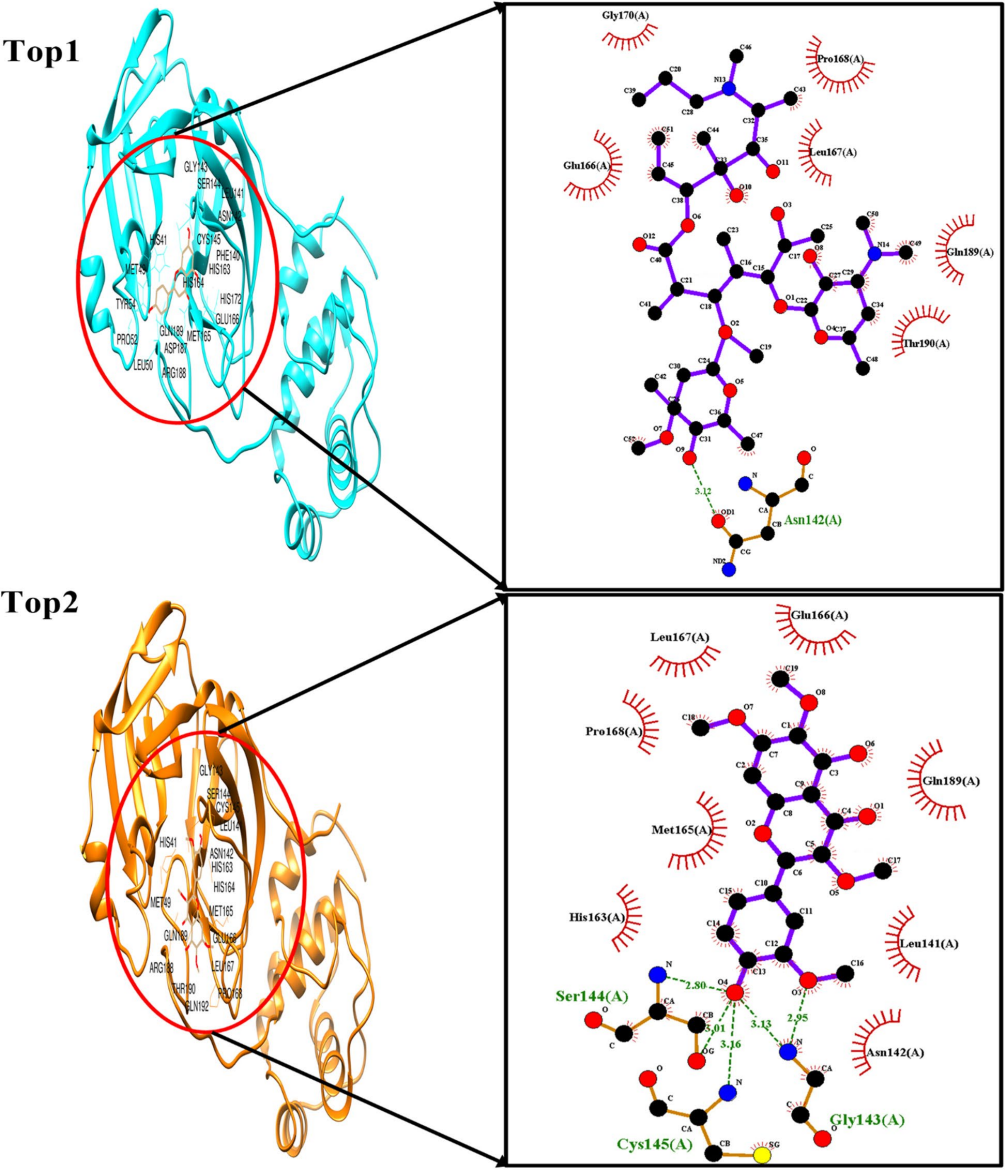
Top1- Interaction of azithromycin with receptor, Top2-Interaction of chrysoplenetin with receptor

The docking results are analyzed by Discovery Studio [43] based on the number of hydrogen bonds, number of hydrophobic interactions, steric interactions and number of interacting amino acids. Figure 3 shows the interaction of azithromycin and chrysoplenetin with the receptor. Azithromycin has formed only one hydrogen bond and ten hydrophobic interactions whereas chrysoplenetin forms seven hydrogen and nine hydrophobic interactions. Table 14 gives the details of this interaction. The binding site of the protease with previously reported inhibitors has been reinvestigated during the interaction of chrysoplenetin. It has been inferred that chrysoplenetin binds to the same residues sites where previously reported inhibitors has been found active [44–46].

**Table 14:** Docking analysis comparison

S.No	Ligand Name	Binding Energy (Kcal/mol)	No.of Hydrogen Bonds	Hydrogen Bonding		Hydrophobic Bonding
				**Amino Acids**	**Distance**	
**1**	**(Top1) Azithromycin**	-6.8	1	O-Thr190-O3	2.90	Cys145 Gly143 Ser144 Leu141 Phe140 Asn142 Glu166 Met165 His164 Gln189
**2**	**(Top2) Chrysoplenetin**	-7.7	7	OG-Ser46-O4 N-Gly143-O5 N-Gly143-O2 N-Ser144-O2 OG-Ser144-O6 NE2-His163-O8 N-Glu166-O7	3.35 2.96 2.81 3.23 3.01 3.04 2.88	Asn142 Cys145 Thr25 Thr24 Thr26 Met49 Leu141 Phe140 Met165

#### Molecular dynamics simulations

**Fig. 4:**
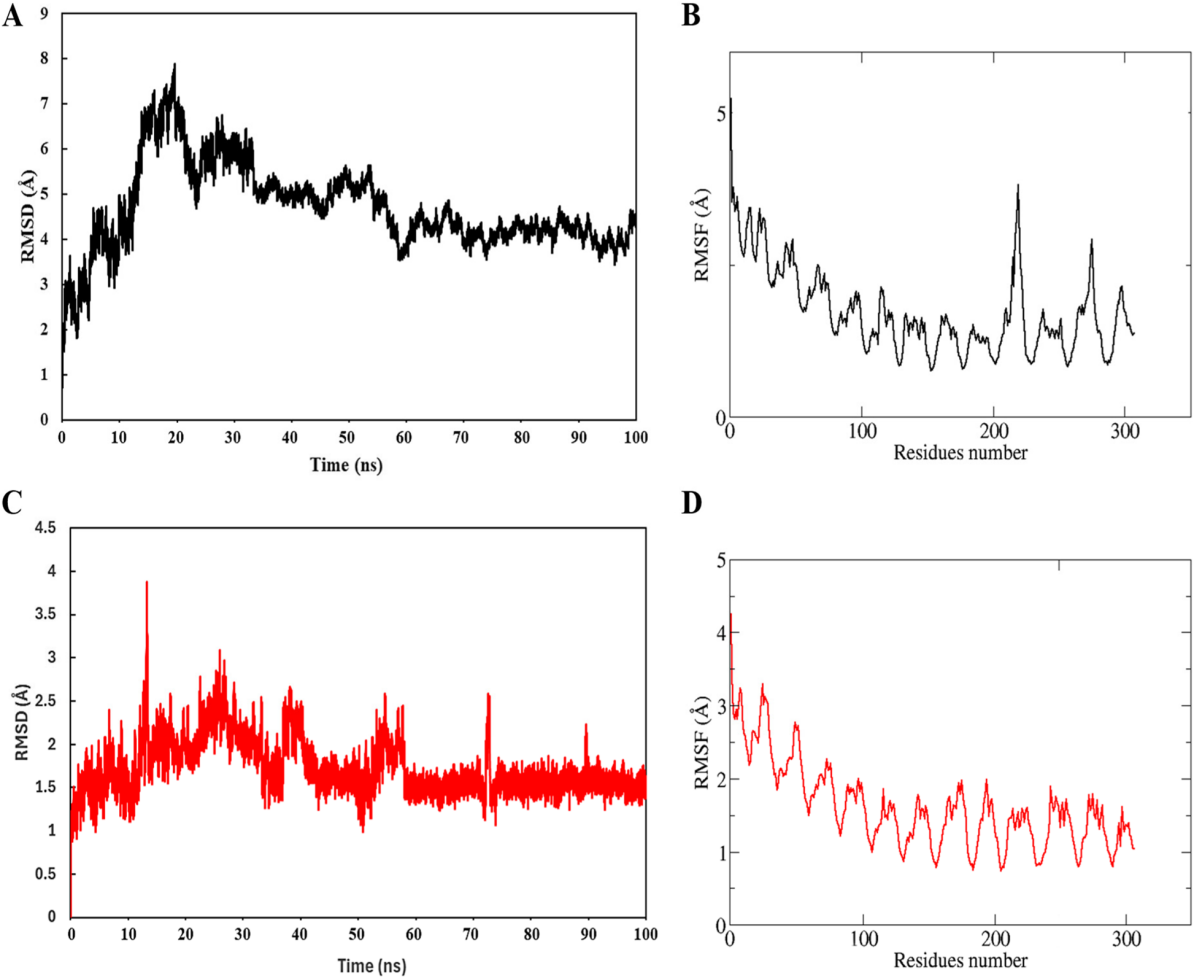
**A** Top1 depicts the RMSD and (**B**) RMSF plot of the C-alpha atoms of ligand-bound proteins throughout time interval. The figures suggest that the protein in the complex with Tocofersolan attained stability at 37 ns till the end of the simulations. Whereas this has been validated in the RMSF graph which attains stability of the system with few minor fluctuations at some loop region residual sites especially from 200–300 residual sites. This is followed by the B-Top2 figure, depicting the stable RMSD with a minor deviation at 20 ns showing a max RMSD of 3.8 Å. **D** depicting the RMSF of Top2 complex system with fluctuation at surface residues at the time scale of 100 ns

Protein conformational dynamics are the most important aspect of its function. The functional information of a protein molecule is contained in its structure. The structure must be thoroughly examined to comprehend its functional variability [47]. MD simulation using AMBER was utilized in this work to investigate the conformational component of protein–ligand interactions. RMSD depicts the backbone analysis and Cα atom dynamics of a docked protein during a 100 ns time for both complexes. A high fluctuation at 20-30 ns was observed with a maximum value was observed in Top1 (Azithromycin) and in Top2 (Chrysoplenetin), this has been depicted among 15-55 ns and the stability for the rest of the time scale has been observed for the rest of the simulation time interval. Herein, Fig. 4 (A) shows that the average RMSD value for docked protein was 4.69 Å, with a maximum peak of 7.87 Å. The RMSD graph does not support any dramatic domain alterations within the protein–ligand complex's structural framework. Whereas the average RMSD value for the Top2 complex observed was 1.71 Å with a maximum RMSD of 3.87 Å. Trajectory analysis was utilized to identify the protein substructures that generated the RMSF trend. This is followed by the RMSF value against both complex, where the average RMSF value recorded for Top1 is 1.7 Å with a maximum value of 5.2 Å and minimum value of 0.7 Å. The RMSD graph revealed substantial structural changes at 15 ns, 25 ns, 45 ns, and 65 ns, after which the protein stabilized. Figure 4 depicts the structural changes that occurred in the docked protein complex at various periods. RMSF quantifies the flexibility and variation of Cα residue structures across time. The average RMSF for the Top2 complex of docked protein for 100 ns was 1.61 Å, with a high of 4.2 Å, and significant changes at residues 76,103, 203 to 279, as well as at the end of the graph for residues 518 and 599. These were largely protein-loop areas where no structural deviation has been observed but shows fluctuations at RMSF level.

#### SASA analysis

**Fig. 5:**
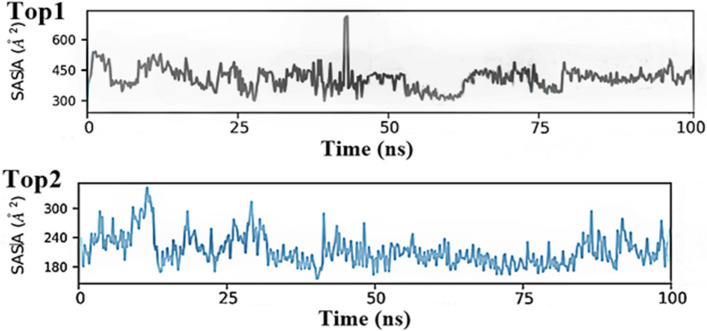
Depicting the dynamics of the Solvent surface residues of both complex system during the time scale of 100 ns

We determined the solvent accessible surface area (SASA) of residues that were linked to significant Drug-Target interaction or catalytic activity, owing to intriguing findings from RMSD and RMSF analysis. When comparing the Top1 to Top2 complex system, the SASA profile (Fig. 5) clearly shows the reduction in the SASA of the critical residues. The enzymatic activities of targeted proteins are inhibited in both systems because of the limited accessibility to critical residues inside complex systems, hence reducing the likelihood of complex interactions. According to the SASA analysis, there must have been some conformational changes in the protein surface, or the amino acid residue moved from the accessible area to the buried region because of complex formation. Stability of the key residues has been monitored throughout the simulation time intervals at the time scale of 100 ns. The average value recorded for Top1 complex system was 325.56 Å^2^ and 191.74 Å^2^ respectively offering a possible dynamics and stability of the system.

#### Hydrogen bond analysis

Hydrogen bonds play an important role during simulation reflecting the stability of the system. This was observed by applying the amber16 tools by giving all the frames of simulation time intervals. It has been inferred that Top1 complex system shows hydrogen bonds among the atom of the residues that include OD1 of Asn142 with O9 of ligand atom. This bond was continuously interacting with the residue till 100 ns time intervals with some minor breakage. This was followed by other hydrogen bonds i.e. Asn142-OD1-LIG-H10 and Glu-O-LIG-H111. Thus, making the Top1 in a stable position inside the active site. On other side the Top2 ligand have a strong hydrogen bonds interaction that includes Gly43 interacting the O3 and O4 atom of the inhibitor molecule followed by Cys145 AND Ser 144 of the target residues.

#### Binding free energies analysis

**Fig. 6:**
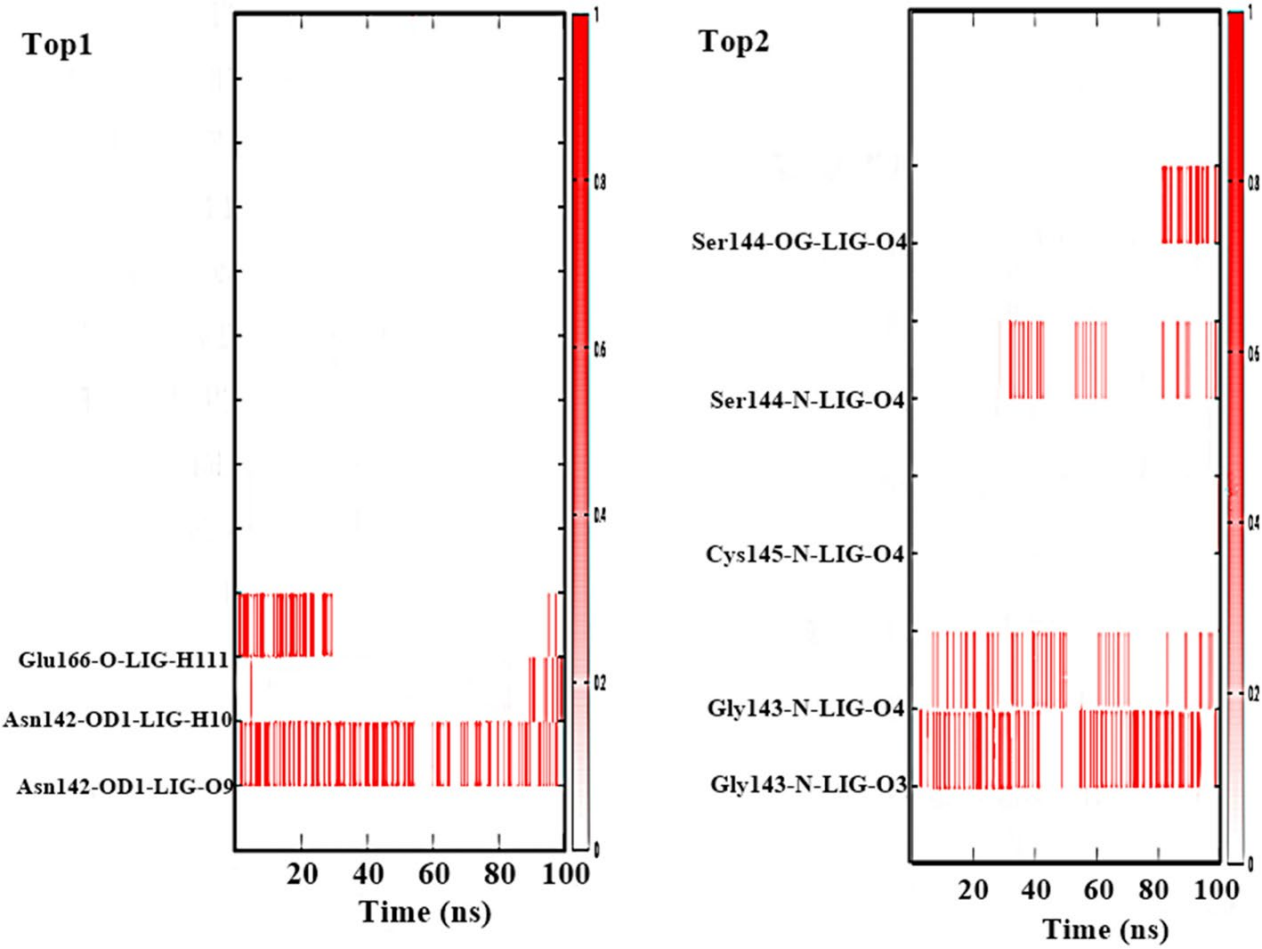
Top1 and Top2 complex depicting the hot spot residues involved in hydrogen bonds during the simulation time intervals after 100 ns time scale

The total binding free energy and complex stability were mostly determined by the energy terms i.e. van der Waals (Evdw), electrostatic (Eele), and solvation (Esolvation), among other energy factors that contributed to the drug-target binding energy. Because end point free-energy modelling techniques, such as MM/PBSA, are known to provide more accurate results than docking binding energy, these were used to investigate the binding free energies of both complexes. Table 15 provides an overview of the binding free energy of both compound complexes. After analysis, the average binding free energy of the Top1 complexes was found to be -45.5898 kcal/mol in MMGBSA and -42.875 kcal/mol during MMPBSA approach. Figure 6 also provide the other binding energies factor which infers the complex's electrostatic (-10.8859 kcal/mol), van der Waals (vdW) (-50.56 kcal/mol), polar surface (-7.1783 kcal/mol) nonpolar surface (-5.678 kcal/mol).Whereas, the Top2 complex system depicting the total binding free of (-44.2875 kcal/mol) for MMGBSA and (-43.376 kcal/mol), with electrostatic energy of (-11.0774 kcal/mol), van der Waals (vdW) (-57.1607 kcal/mol), polar surface energy (-8.8117 kcal/mol) and nonpolar surface energy (–5.6832 kcal/mol). Herein, for the Top1 and Top2 complex system the calculations of free energy showed that vdW energy plays a significant role in binding free energy, thus indicating the Top2 complex system shows a low binding energies with high affinity during the time intervals of 100 ns, thus helps the complex become transiently stable.

**Table 15:** Binding free energies calculation of both Top1 and Top2 after simulation time intervals of 100 ns

Top1-MMGBSA	
VDWAALS	-50.56
EEL	-10.8859
ESURF	-7.1783
DELTA TOTAL	-45.5898
Top1-MMPBSA	
VDWAALS	-50.56
EEL	-10.8859
ENPOLAR	-5.678
DELTA TOTAL	-42.875
Top2-MMGBSA	
VDWAALS	-57.1607
EEL	-11.0774
ESURF	-8.8117
DELTA TOTAL	-44.2875
Top2-MMPBSA	
VDWAALS	-57.1607
EEL	-11.0774
ENPOLAR	-5.6832
DELTA TOTAL	-43.376

#### PCA analysis

**Fig. 7:**
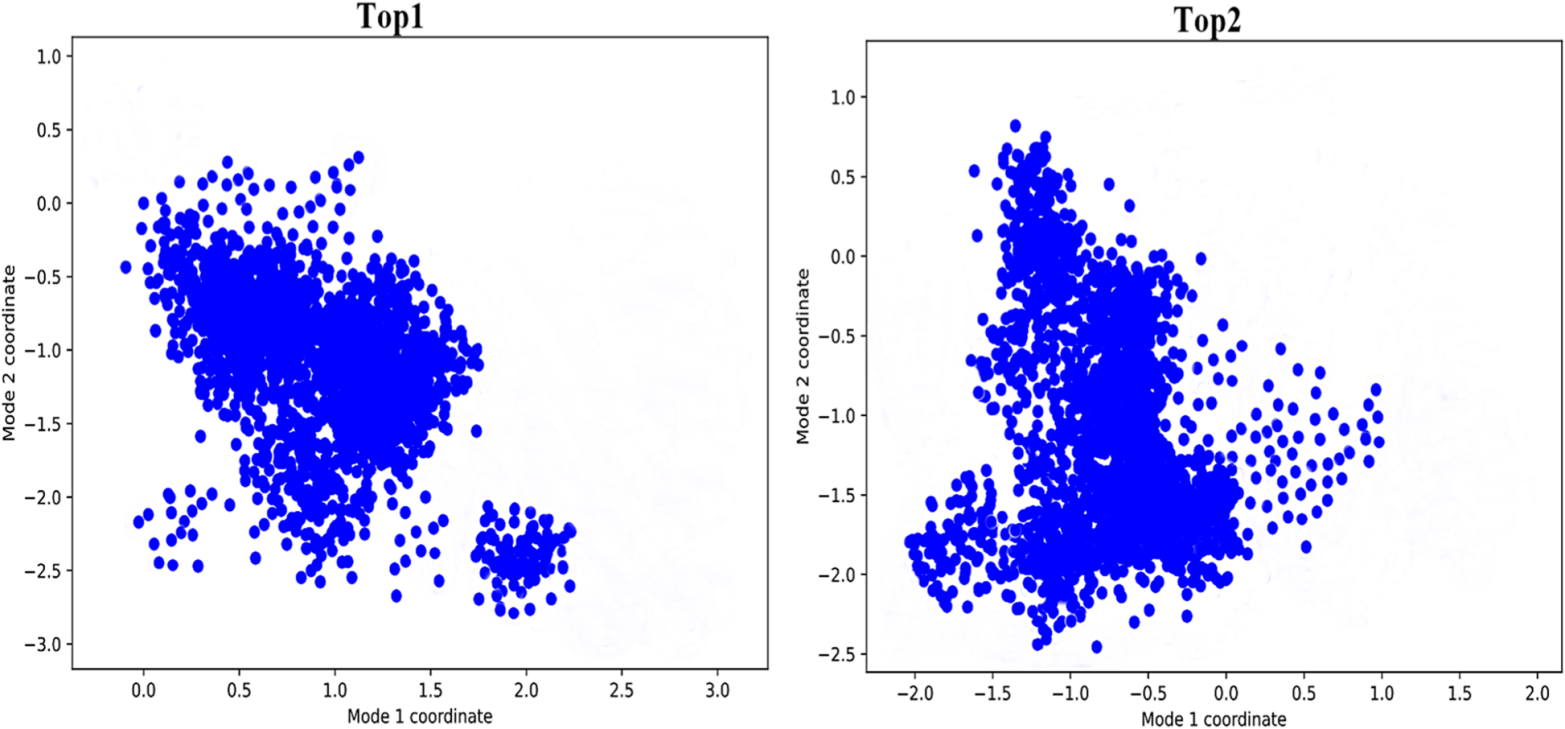
PCA analysis. Top1 and Top2 complex systems show the movement of residues in comparison at the end of the simulation time intervals

To deduce the mechanical characteristics, such as structural movements and variations, of both complexes Principal Component Analysis (PCA) analysis was performed. Using vectorial representations, a collection of eigenvectors representing the motion of each individual component was obtained from the MD trajectories as shown in Fig. 7. Results evaluated shows the mobility of protein residues in both the system and it has been analyzed that both the drug bind to the active residues shows a slight deviation and alteration during the simulation time intervals. Clusters residues in the Top1 and Top2 system moving towards the lower state energies with drug molecules moving deep inside the active cavity of the target protein. Herein, Top2 complex system shows more deviation on x-axis as compared to Top1 which depicts the residues are moving towards high lower state level making the system more stable.

## Discussion

With the emergence of the human SARS Coronavirus 2, many questions related to the evolution and introduction of the virus in the human race were raised. The biological community was indulged in finding answers to the reservoirs of the virus, its spread and its effect on the human race [48]. It was found that upon transcription the beta coronavirus produces 800KD polypeptide, which is cleaved by papain-like protease and 3-chymotrypsin-like protease to generate various non-structural proteins that are involved in viral replication [49]. The main protease protein having an essential role in the replication of viruses served as an amazing drug target site [5]. Many drugs were used against M^pro^ and few vaccines were developed for the inhibition of virus replication. Azithromycin has been commonly used in Pakistan and other countries. It has shown vital results during respiratory, chronic inflammatory disorders and bronchiolitis [12] for this reason it is used as a control. Medicinal plants have shown therapeutic properties, *Artemisia annua* is a herb used against malaria and other fevers. In Madagascar, a drink was prepared with an infusion of *Artemisia annua* to cure Covid-19 [14]. For this purpose, the plant was exploited, and 25 active constituents were selected to be docked against the main protease.

CB-dock a blind docking tool was used. Docking focuses on cavity binding so that ratio of accuracy is higher [50]. For performing docking the 3D structure of a protein in pdb format and the 3D structure of the ligand in sdf format was uploaded. The pose with minimum vina scores in KJ/mol was selected [51]. The docking results were visualized in PyMol and the 2D representation was generated in LigPlot. After studying the ADMET properties of the ligands and passing them from the Lipinski Rule, 9 out of 25 ligands were selected which were quercetin, rutin, casticin, chrysoplenetin, apigenin, artemetin, artesunate, scopolin and sitogluside. After their analysis, chrysoplenetin was selected as the lead compound and was compared with the standard drug azithromycin.

Predictions of ADMET using in silico methods are essential for drug development, but they are subject to constraints including dependence on high-quality data, the application of the model, comprehension of intricate biological processes, variability between individuals, and difficulties in precisely predicting toxicity. The aforementioned constraints require enhancements in predicting methodologies and the incorporation of other scientific methodologies [52]. Hrein, Azithromycin was shown to break the molecular weight and H-bond acceptor rule of Lipinski [24] whereas chrysoplenetin follows all the rules. Azithromycin was proved to be hepatotoxic when toxicity comparison was done by pkcsm. In the *T.pyriformis* (model 8) toxicity test, azithromycin was proved to be somewhat toxic. The intestinal absorption of azithromycin is also less than chrysoplenetin. Furthermore, Azithromycin showed a binding score of -6.8 kcal/mol forming only one hydrogen bond and ten hydrophobic bonds whereas chrysoplenetin showed a score of -7.7 kcal/mol forming seven hydrogen bonds and nine hydrophobic bonds. According to MD simulations, the drug receptor complex became stable in the physiochemical environment as its shape altered over time. Despite minor alterations inside chain and loop mobility, the inhibitor remained stable. The structural stability of the docked complex following simulation studies implies that the chosen ligand might be a viable lead chemical. Results were quite interesting as the protein in complex with chrysoplenetin attained high stability throughout the simulations time interval. Where a little change has been recorded in the RMSF. Binding free energies, SASA analysis, Hydrogen bond analysis and PCA analysis has shown a high ground stability of chrysoplenetin in complex with a target protein during the simulation time interval of 100 ns. All these studies showed that chrysoplenetin is a better drug candidate against the reported protein structure. Herein, Molecular dynamics (MD) simulations provide a comprehensive depiction of the fluctuations and conformational changes seen in biological systems by replicating the actual motions of atoms and molecules over a period of time. These parameters, including stability, binding affinity, and conformational alterations, play a crucial role in establishing the biological significance of the complex system. Such knowledge provided is of great use in furthering our comprehension of biological molecules and their complexes, as well as in the development of novel pharmaceuticals and treatments.

## Conclusions

Covid-19 has created massive problems for the human race. M^pro^ was revealed to be an active drug target. Some plants were exploited for their efficiency against M^pro^. 25 ligands from the plant *Artemisia annua* showing antiviral properties were selected. Based on the inhibition effect involving hydrogen bonding and hydrophobic interaction, chrysoplenetin was selected as a lead compound which was then compared with the standard drug azithromycin. The inhibition process is explained by how the inhibitor binds and how the target protein acts as a catalyst. Likewise, the structure dynamics of the docked protein reveal useful information that can be used to improve the drug's effectiveness against the comparison between these two shows that chrysoplenetin can be better and more effective against M^pro^. This research contributes to the global effort to combat SARS-CoV-2 and provides a foundation for addressing future viral threats by emphasizing the potential of chrysoplenetin as a therapeutic agent. The recognition of chrysoplenetin as a highly effective inhibitor presents opportunities for additional comprehensive investigations, such as clinical trials, to ascertain its safety and efficacy in human subjects. Furthermore, the approaches and insights acquired have the potential to expedite the identification of innovative inhibitors targeting new viral proteases, thereby enhancing our readiness for prospective pandemics.

### Supplementary Information

Supplementary Materials 1:** Table S1. **Applicability of Lipinski rule.** Table S2 **Selected ligands with structural information.** Table S3 **Absorption properties of the ligands and standard drug.** Table S4 **Distribution properties of the ligands and standard drug.** Table S5** Lipinski’s Rule Comparison.

## Data Availability

Data will be available on suitable request to corresponding author. (https://www.rcsb.org/structure/6lu7).
