# Supplementary material for: Phytoconstituents of Artemisia annua as potential inhibitors of SARS CoV2 main protease: an in silico study
Source: BMC Infect Dis. 2024 May 15;24:495. doi: 10.1186/s12879-024-09387-w (PMC11094927; doi:10.1186/s12879-024-09387-w)
Supplement: Supplementary file 1 — Supplementary Materials 1: Table S1. Applicability of Lipinski rule. Table S2 Selected ligands with structural information. Table S3 Absorption properties of the ligands and standard drug. Table S4 Distribution properties of the ligands and standard drug. Table S5 Lipinski’s Rule Comparison. [file 12879_2024_9387_MOESM1_ESM.docx]

Supplementary materials

**Phytoconstituents of Artemisia Annua as potential inhibitors of SARS CoV2 main protease: An *in silico* study**

**Table S1** Applicability of Lipinski rule

| **S. No** | **Ligand** | **Log P-value** | **Molecular Weight**  **g/mol** | **H-bond Acceptor** | **H-bond Donor** |
| --- | --- | --- | --- | --- | --- |
| **1.** | Alpha-pinene | 2.9987 | 136.23 | **0** | **0** |
| **2.** | Beta pinene | 2.9987 | 136.23 | **0** | **0** |
| **3.** | Carvone | 2.4879 | 150.22 | **1** | **0** |
| **4.** | Myrtenol | 1.9711 | 152.23 | **1** | **1** |
| **5.** | Quinic acid | -2.3214 | 192.17 | **5** | **5** |
| **6.** | Caffeic acid | 1.1956 | 180.16 | **3** | **3** |
| **7.** | Quercetin | 1.988 | 302.23 | **7** | **5** |
| **8.** | Rutin | -1.6871 | 610.5 | **16** | **10** |
| **9.** | Apigenin | 2.5768 | 270.24 | **5** | **3** |
| **10.** | Chrysoplenetin | 2.9056 | 374.3 | **8** | **2** |
| **11.** | Arteannunin b | 2.4518 | 248.32 | **3** | **0** |
| **12.** | Artemisinin | 2.3949 | 282.33 | **5** | **0** |
| **13.** | Scopoletin | 1.5072 | 192.17 | **4** | **1** |
| **14.** | Scoparone | 1.8102 | 206.19 | **4** | **0** |
| **15.** | Artemisnic acid | 3.6458 | 234.33 | **1** | **1** |
| **16.** | Deoxyartemisnin | 2.4633 | 266.33 | **4** | **0** |
| **17.** | Artemetin | 3.2086 | 388.4 | **8** | **1** |
| **18.** | Casticin | 2.9056 | 374.3 | **8** | **2** |
| **19.** | Sitogluside | 5.849 | 576.8 | **6** | **4** |
| **20.** | Beta-sitosterol | 8.0248 | 414.7 | **1** | **1** |
| **21.** | Dihydroartemisnin | 2.1867 | 284.35 | **5** | **1** |
| **22.** | Scopolin | -1.0197 | 354.31 | **9** | **4** |
| **23.** | Artemether | 2.8408 | 298.37 | **5** | **0** |
| **24.** | Artemotil | 3.2309 | 312.4 | **5** | **0** |
| **25.** | Artesunate | 2.6024 | 384.4 | **7** | **1** |

**Table S2** Selected ligands with structural information

| S. No. | Name | Molecular Formula | Molecular Weight g/mol | Structure |
| --- | --- | --- | --- | --- |
|  | alpha-pinene | [C_10_H_16_](https://pubchem.ncbi.nlm.nih.gov/#query=C10H16) | 136.23 | 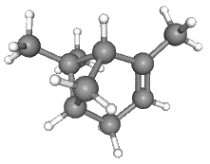 |
|  | beta pinene | [C_10_H_16_](https://pubchem.ncbi.nlm.nih.gov/#query=C10H16) | 136.23 | 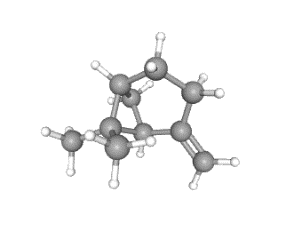 |
|  | Carvone | [C_10_H_14_O](https://pubchem.ncbi.nlm.nih.gov/#query=C10H14O) | 150.22 | 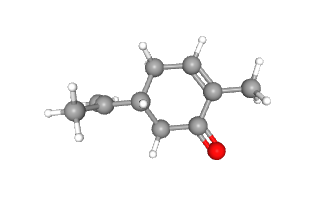 |
|  | Myrtenol | [C_10_H_16_O](https://pubchem.ncbi.nlm.nih.gov/#query=C10H16O) | 152.23 | 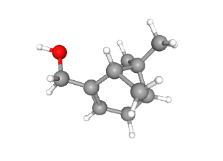 |
|  | quinic acid | C7H12O6 | 192.17 | 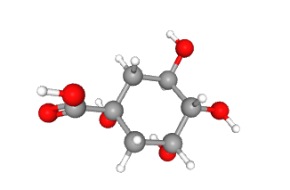 |
|  | Caffeic acid | [C_9_H_8_O_4_](https://pubchem.ncbi.nlm.nih.gov/#query=C9H8O4) | 180.16 | 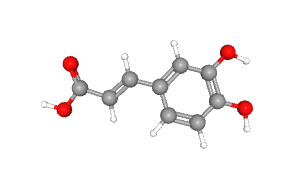 |
|  | Quercetin | [C_15_H_10_O_7_](https://pubchem.ncbi.nlm.nih.gov/#query=C15H10O7) | 302.23 | 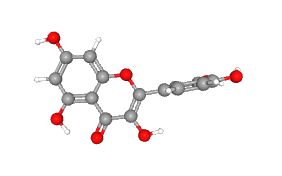 |
|  | Rutin | [C_27_H_30_O_16_](https://pubchem.ncbi.nlm.nih.gov/#query=C27H30O16) | 610.5 | 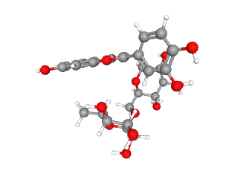 |
|  | Apigenin | [C_15_H_10_O_5_](https://pubchem.ncbi.nlm.nih.gov/#query=C15H10O5) | 270.24 | 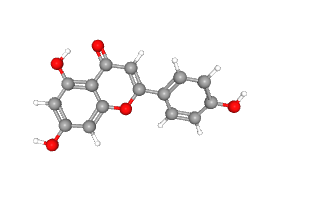 |
|  | chrysoplenetin | [C_19_H_18_O_8_](https://pubchem.ncbi.nlm.nih.gov/#query=C19H18O8) | 374.3 | 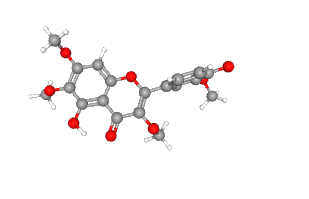 |
|  | Arteannunin b | [C](https://pubchem.ncbi.nlm.nih.gov/#query=C15H20O3)_[15](https://pubchem.ncbi.nlm.nih.gov/#query=C15H20O3)_[H](https://pubchem.ncbi.nlm.nih.gov/#query=C15H20O3)_[20](https://pubchem.ncbi.nlm.nih.gov/#query=C15H20O3)_[O](https://pubchem.ncbi.nlm.nih.gov/#query=C15H20O3)_[3](https://pubchem.ncbi.nlm.nih.gov/#query=C15H20O3)_ | 248.32 | 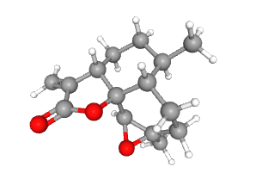 |
|  | artemisinin | [C_15_H_22_O_5_](https://pubchem.ncbi.nlm.nih.gov/#query=C15H22O5) | 282.33 | 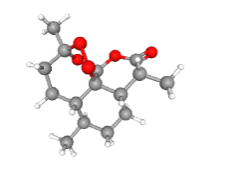 |
|  | Scopoletin | [C_10_H_8_O_4_](https://pubchem.ncbi.nlm.nih.gov/#query=C10H8O4) | 192.17 | 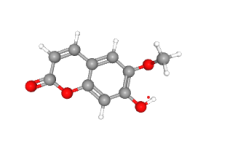 |
|  | Scoparone | [C_11_H_10_O_4_](https://pubchem.ncbi.nlm.nih.gov/#query=C11H10O4) | 206.19 | 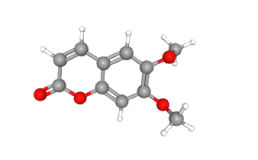 |
|  | Artemisnic acid | [C_15_H_22_O_2_](https://pubchem.ncbi.nlm.nih.gov/#query=C15H22O2) | 234.33 | 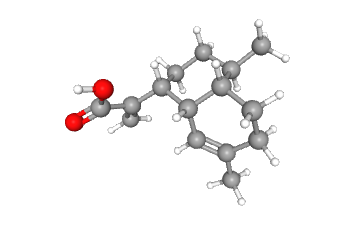 |
|  | Deoxyartemisnin | [C_15_H_22_O_4_](https://pubchem.ncbi.nlm.nih.gov/#query=C15H22O4) | 266.33 | 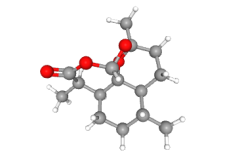 |
|  | Artemetin | [C_20_H_20_O_8_](https://pubchem.ncbi.nlm.nih.gov/#query=C20H20O8) | 388.4 | 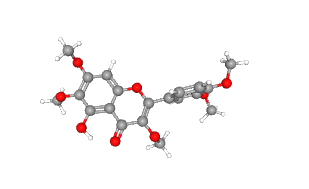 |
|  | Casticin | [C](https://pubchem.ncbi.nlm.nih.gov/#query=C19H18O8)_[19](https://pubchem.ncbi.nlm.nih.gov/#query=C19H18O8)_[H](https://pubchem.ncbi.nlm.nih.gov/#query=C19H18O8)_[18](https://pubchem.ncbi.nlm.nih.gov/#query=C19H18O8)_[O](https://pubchem.ncbi.nlm.nih.gov/#query=C19H18O8)_[8](https://pubchem.ncbi.nlm.nih.gov/#query=C19H18O8)_ | 374.3 | 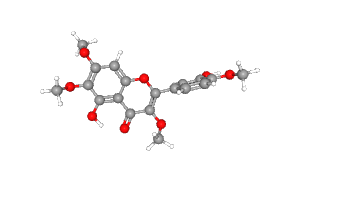 |
|  | Sitogluside | [C_35_H_60_O_6_](https://pubchem.ncbi.nlm.nih.gov/#query=C35H60O6) | 576.8 | 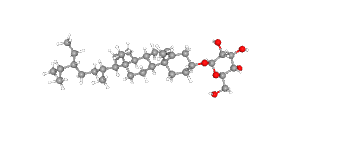 |
|  | beta sitosterol | [C_29_H_50_O](https://pubchem.ncbi.nlm.nih.gov/#query=C29H50O) | 414.7 | 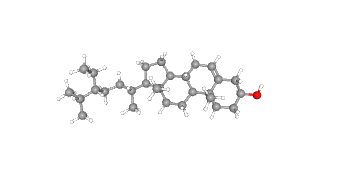 |
|  | Dihydroartemisnin | [C_15_H_24_O_5_](https://pubchem.ncbi.nlm.nih.gov/#query=C15H24O5) | 284.35 | 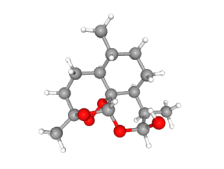 |
|  | Scopolin | [C_16_H_18_O_9_](https://pubchem.ncbi.nlm.nih.gov/#query=C16H18O9) | 354.31 | 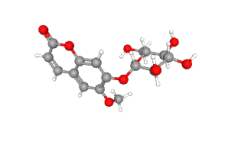 |
|  | Artemether | [C_16_H_26_O_5_](https://pubchem.ncbi.nlm.nih.gov/#query=C16H26O5) | 298.37 | 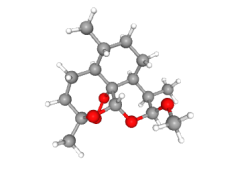 |
|  | Artemotil | [C_17_H_28_O_5_](https://pubchem.ncbi.nlm.nih.gov/#query=C17H28O5) | 312.4 | 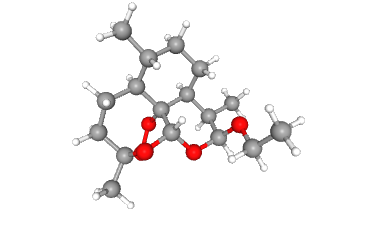 |
|  | Artesunate | [C_19_H_28_O_8_](https://pubchem.ncbi.nlm.nih.gov/#query=C19H28O8) |  | 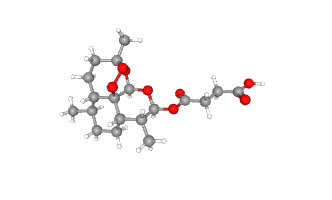 |

**Table S3** Absorption properties of the ligands and standard drug

| S.No. | Ligands | Water Solubility  Log mol/L | CaCO_2_ Solubility  log Papp | Intestinal Absorption  (human) | Skin Permeability | P-glycoprotein substrate | P-glycoprotein I inhibitor | P-glycoprotein II inhibitor |
| --- | --- | --- | --- | --- | --- | --- | --- | --- |
| 1 | Quercetin | -2.925 | -0.229 | 77.207 | -2.735 | Yes | No | No |
| 2 | Rutin | -2.892 | -0.949 | 23.446 | -2.735 | Yes | No | No |
| 3 | Casticin | -3.599 | 1.39 | 96.91 | -2.744 | Yes | No | Yes |
| 4 | Chrysoplenetin | -3.605 | 1.393 | 99.856 | -2.743 | Yes | No | Yes |
| 5 | Apigenin | -3.329 | 1.007 | 93.25 | -2.735 | Yes | No | No |
| 6 | Artemetin | -4.326 | 1.424 | 100 | -2.747 | Yes | Yes | Yes |
| 7 | Artesunate | -3.097 | 0.863 | 72.19 | -2.735 | Yes | No | No |
| 8 | Scopolin | -2.21 | 0.377 | 48.119 | -2.822 | Yes | No | No |
| 9 | Sitogluside | -4.741 | 0.472 | 79.677 | -2.748 | Yes | Yes | Yes |

**Table S4** Distribution properties of the ligands and standard drug

| **S. No** | **Ligand** | **VDss (human)**  **L/Kg** | **Fraction unbound (human)** | **BBB Permeability**  **logBB** | **CNS Permeability**  **logPS** |
| --- | --- | --- | --- | --- | --- |
| **1.** | Quercetin | -1.559 | 0.20 | -1.098 | -3.065 |
| **2.** | Rutin | 1.663 | 0.187 | -1.899 | -5.178 |
| **3.** | Apigenin | 0.822 | 0.147 | -0.734 | -2.061 |
| **4..** | Chrysoplenetin | -0.161 | 0.103 | -1.043 | -3.226 |
| **5.** | Artemetin | -0.244 | 0.123 | -1.152 | -3.156 |
| **6.** | Casticin | -0.176 | 0.103 | -1.053 | -3.209 |
| **7.** | Sitogluside | -1.163 | 0.078 | -0.785 | -3.021 |
| **8.** | Scopolin | -0.611 | 0.397 | -1.286 | -3.954 |
| **9.** | Artesunate | 0.172 | 0.36 | -0.954 | -3.039 |
| **10.** | Azithromycin (Standard drug) | -0.214 | 0.512 | -1.857 | -3.777 |

**Table S5** Lipinski’s Rule Comparison

| **S. No.** | Name of Compound | Log P-value | Molecular Weight g/mol | H-bond acceptor | H-bond donor |
| --- | --- | --- | --- | --- | --- |
| **1.** | Azithromycin | 1.9007 | 748.996 | 14 | 05 |
| **2.** | Chrysoplenetin | 2.9056 | 374.3 | 8 | 2 |
